# Efficacy and safety of transvenous lead extraction in the Chinese octogenarian patients

**DOI:** 10.1002/clc.23629

**Published:** 2021-06-17

**Authors:** Xu Zhou, Feng Ze, Xuebin Li, Bin Wang

**Affiliations:** ^1^ Department of Cardiology The First Affiliated Hospital of Shantou University Medical College Shantou Guangdong China; ^2^ Department of Cardiac Electrophysiology Peking University People's Hospital Beijing China

**Keywords:** octogenarians, pacemaker, procedure outcomes, transvenous lead extraction

## Abstract

**Background:**

Managing elderly patients with infection or malfunction deriving from a cardiac implantable electronic device (CIED) may be challenging. We report the safety and effectiveness of transvenous lead extraction in Chinese octogenarian patients.

**Hypothesis:**

Transvenous lead extraction can be performed safely and successfully in the Chinese octogenarians.

**Methods:**

We retrospectively identified all patients who underwent TLE at our institution between March 2013 and January 2021. This population was divided into the following two groups based solely on age: octogenarians and non‐octogenarians. These two groups were compared on the basis of several characteristics and clinical outcomes.

**Results:**

Consecutive 1106 patients were analyzed, including 184 (16.6%) octogenarians and 922(83.4%) non‐octogenarians. The octogenarians presented more comorbidities. A total of 378 leads and 2004 leads were removed from the patients in the octogenarian group and non‐octogenarian group, respectively. The mean lead implant duration was 6.1 ± 5.1 and 5.8 ± 4.6 years for octogenarians and non‐octogenarians (p = 0.296). The majority of the patients in both groups underwent TLE through the femoral approach (67.5% vs 61.6%, p = 0.14). The complete procedure success rate was similar between the octogenarian and non‐octogenarian group (96.7% vs.95.3%, p = 0.39). There were no differences with respect to the proportion of minor, major complications and deaths. Long‐term mortality (median time 3.3 years) after TLE in octogenarians was significantly higher compared with younger individuals.

**Conclusions:**

At experienced centers, transvenous lead extraction can be performed safely in Chinese octogenarians with procedural success rate and complication rate comparable to younger individuals.

## 1 INTRODUCTION

The number of elderly patients with cardiovascular implantable electronic devices (CIEDs) remains still growing due to the global population aging, which results in increased subpopulation of elderly patients with late complications, such as infections or lead malfunction.^1^, ^2^ Transvenous lead extraction (TLE) is the cornerstone in the management of CIED–related adverse events.^3^ There is a considerable controversy regarding TLE safety in elderly patients due to their potentially worse general condition, more concomitant diseases and additional difficulties in sedation or analgesia. According to the prospective multicenter ELECTRa (European Lead Extraction Controlled) study on TLE procedures, the age above 68 years is one of the predictors of increased all‐cause mortality during hospitalization.^4^ However, a few single center studies from USA and European countries showed that laser‐assisted lead extraction in elderly patients (>80 years) was safety and effective.^5^, ^6^, ^7^, ^8^ There is a scarcity of data for the Asian population. The Asian population presents lower body mass index, which is known as a risk factor for major adverse events associated with transvenous lead extraction.^9^, ^10^, ^11^ Different from the laser technique widely used in western countries, the femoral approach, with less cost, has long been used in a large number of medical centers in China. There have been no reports on outcomes of femoral approach procedure in elderly patients. The present study aimed to assess the effectiveness and safety of TLE and long‐term survival in the Chinese octogenarian patients at a single high‐volume tertiary cardiovascular referral center.

## 2 METHODS

### 2.1 Study population and data collection

We performed retrospective analysis of data achieved from 1106 consecutive patients undergoing TLE with infective or non‐infective indications between March 2013 and January 2021. Baseline clinical characteristics, procedural details, and survival data were obtained from the review of the medical records, institutional databases, and phone interview. Patients were divided into two groups based on age: Group 1 included patients younger than 80 years and group 2 patients aged 80 or over. Patient and lead characteristics, indications for extraction, associated medical conditions, and clinical outcomes were compared between these two groups. The study was approved by an ethics committee, and complied with the Declaration of Helsinki.

The indications for transvenous lead extraction were also defined by the guidelines.^12^The device‐related infections were subdivided as follows: (1) isolated pocket infection (local signs of inflammation, with negative blood cultures), (2) bacteremia (positive blood cultures with or without systemic infection symptoms and signs), (3) pocket infection with bacteremia (local signs of pocket infection and positive blood cultures, without lead or valvular vegetations), (4) pocket infection with lead/valvular endocarditis (local signs of pocket infection and positive blood cultures and lead or valvular vegetations), (6) device‐related endocarditis without pocket infection (bacteremia with or without lead or valvular vegetations, and without local signs of pocket infection). The non‐infectious indication included: (1) lead dysfunction (lead fracture or insulation failure resulting in issues with lead impedance, sensing or capture), (2) abandoned functional leads (a functional lead may no longer be required and may be extracted to reduce the intravascular lead burden in order to avoid future issues), (3) lead‐related complications (leads may be functional but cause complications for which extraction may be indicated, such as thromboembolic events, superior vena cava syndrome, arrhythmias, perforation, severe tricuspid regurgitation), (4) other indications (access to magnetic resonance imaging, radiation therapy).

### 2.2 Lead extraction procedure: tools and approaches

The anesthesia method and the use of particular extraction tools were at the discretion of the operator. Lead extraction procedures were performed under general anesthesia, conscious sedation or local anesthesia. Transesophageal echocardiography was performed in patients who.

were given general anesthesia. Both superior approach and femoral approach were used at our institute. Simple manual traction, consisting of the removal of a lead using manual tools such as stylets (locking and nonlocking) and fixation screw retraction clips, was applied first. If the lead remained immobile in the venous system after a few minutes, the following three techniques were adopted: (1) femoral approach with Needle's Eye Snare (Cook Medical Inc.), or the Amplatz Goose Neck Snare Kit (Covidien, USA) (2) mechanical sheath extraction, consisting of lead removal through a Polypropylene or Teflon dilator sheath (Byrd, Cook Vascular Inc, Leechburg, PA, USA) and using both locking and nonlocking stylets; and (3) laser‐assisted lead extraction, consisting of lead removal through an excimer laser sheath (SLS II, Spectranetics, Colorado Springs, CO, USA) using a locking stylet.

### 2.3 Outcomes

The outcomes of TLE procedure were defined according to the 2017 Heart Rhythm Society Expert Consensus Statement on Cardiovascular Implantable Electronic Device Lead Management and Extraction, and the 2018 European Heart Rhythm Association (EHRA) Expert Consensus Statement on Lead Extraction.^3^, ^12^ Complete procedural success and clinical success were defined as removal of all targeted leads and material, with the absence of any permanently disabling complication or procedure‐related death, or retention of a small part of the lead that did not negatively affect the outcome goals of the procedure, respectively. Procedure failure was defined as inability to achieve either complete procedural or clinical success, or the development of any permanently disabling complication or procedural‐related death. Radiological success (considered for each lead) was defined when the lead was completely removed, partial success when less than a 4 cm of a lead remained in the patient and failure when more than a 4 cm length of a lead was abandoned after a removal attempt.

Major complication was defined as any of the outcomes related to the procedure, which was life‐threatening or resulted in death or any unexpected event that caused persistent or significant disability. Minor complication was defined as any undesired event related to the procedure that required medical intervention or minor procedural intervention to remedy and did not limit persistently or significantly the patient's function, threaten life or cause death. Both major and minor complications were grouped together for each population to create a composite of adverse events, and this was further analyzed.

## 3 STATISTICAL ANALYSIS

The values presented are expressed as mean ± *SD* for continuous variables showing normal distribution, and as frequencies and percentages for categorical data. Continuous variables were compared using independent Student t‐test. Categorical variables were compared using the Chi‐square statistic or Fisher's exact test. A two‐tailed p value of <.05 was considered to indicate statistical significance. Differences in long‐term survival were assessed by Kaplan Meier product limit estimates and tested with the log‐rank test. Data analysis was performed using SPSS (IBM SPSS Statistics for Windows, Version 25.0, IBM Corp, Armonk, NY).

## 4 RESULTS

A total of 1106 patients underwent TLE in our center during the study period. This population was separated into two groups: Group 1 (age < 80) and Group 2 (age ≥ 80). Demographic characteristics of both groups are detailed in Table 1. Group 1 comprised 922 patients (83.4%), with a total of 2004 leads, and Group 2 (age < 80 years) comprised 184 patients (16.6%), with 378 leads. There were more men in Group 2 (77.7% vs. 69.7%, p = .037). Patients in the elderly group had lower BMI (20.7 ± 3.1 kg/m^2^ vs. 22.4 ± 3.3 kg/m^2^, p < .001), lower left ventricular ejection fraction (42.8 ± 16.4% vs. 48.9 ± 12.8%, p < .001) and lower hemoglobin(11.7 ± 2.1 g/dl vs. 13.0 ± 1.8 g/dl, p < .001) than those in the non‐elderly group. The over‐80s presented more comorbidities, such as coronary artery disease (27.7% vs 16.3%, p < .001), atrial fibrillation (19.0% vs 12.6%, p = .027), chronic renal disease (9.8% vs 4.3%, p = .024) and diabetes mellitus (26.1% vs 18.5%, p = .019) and cerebrovascular disease (10.9% vs 3.6%, P < 0.001). Overall morbidity measured with the Charlson Comorbidity Index (CCI) was higher in octogenarians than in younger patients (2.4 ± 1.6 vs. 2.1 ± 1.7, p = .022).

**TABLE 1:** Baseline characteristics of patients

	Age < 80 (*n* = 922)	Age > 80 (*n* = 184)	p value
Age, year	62 ± 13.7	83.8 ± 3.3	<.001
Male sex	643 (69.7)	143 (77.7)	.037
BMI, Kg/m^2^	22.4 ± 3.3	20.7 ± 3.1	<.001
Comorbid conditions			
CAD	150 (16.3)	51 (27.7)	<.001
AF	116 (12.6)	35 (19.0)	.027
HTN	369 (40.0)	82 (44.6)	0.25
CHF	46 (5.0)	15 (8.2)	.086
CRF	40 (4.3)	18 (9.8)	.024
DM	171 (18.5)	48 (26.1)	.019
Cerebrovascular disease	33 (3.6)	20 (10.9)	<.001
Charlson comorbidity index	2.1 ± 1.7	2.4 ± 1.6	.022
LVEF, %	48.9 ± 12.8	42.8 ± 16.4	<.001
Creatinine, μmol/l	90 ± 56	106 ± 32	.003
Hemoglobin, g/dl	13.0 ± 1.8	11.7 ± 2.1	<.001
Antiplatelets	271 (29.4)	56 (30.2)	0.77
Anticoagulants	263 (28.6)	65 (35.6)	.065

*Note*: Data are presented as mean ± *SD* or n (%).

Abbreviations: AF, atrial fibrillation; BMI, body mass index; CAD, coronary artery disease; CHF, chronic heart failure; CRF, chronic renal failure; DM, diabetes mellitus; LVEF, left ventricular ejection fraction.

The most common device for both groups was pacemaker (Table 2). In the octogenarian group, there were more pacemakers (89.2% vs. 81.7%, p = .014) and fewer cardiac resynchronization.

**TABLE 2:** Devices and leads

	Age < 80 (*n* = 922)	Age > 80 (*n* = 184)	p value
Type of devices			
ICD	70 (7.6)	10 (5.4)	0.30
CRT‐D	56 (6.1)	7 (3.8)	0.22
PM	753 (81.7)	164 (89.2)	.014
CRT‐P	43 (4.6)	2 (1.1)	.025
Leads			
Total	2004	378	
Number of total leads per patient	2.2 ± 0.8	2.1 ± 0.7	.064
Mean dwelling time of the leads, year	5.8 ± 4.6	6.1 ± 5.1	0.296
Passive fixation lead	1649 (82.3)	326 (86.2)	0.57
ICD lead	126 (6.3)	17 (4.5)	0.18
Dual coil	96 (4.8)	8 (2.1)	.019
Atrial lead	743 (37.1)	141 (37.3)	0.93
Right ventricular lead	1262 (63.0)	228 (60.3)	0.33
Coronary sinus lead	99 (4.9)	9 (2.4)	.028

*Note*: Data are presented as mean ± *SD* or n (%).

Abbreviations: CRT‐D, cardiac resynchronization therapy with a defibrillator; CRT‐P, cardiac resynchronization therapy with a pacemaker; ICD, implantable cardioverter defibrillator; PM, pacemaker.

therapy‐pacemakers (1.1% vs. 4.6%, p = .025). Complexity of the system was understood as the number of leads in heart before lead extraction. The number of total leads per patient did not differ between the groups (2.2 ± 0.8 vs. 2.1 ± 0.7, p = .064). The mean dwelling time (year) for the octogenarian group was longer than the non‐octogenarian group (6.1 ± 5.1 vs. 5.8 ± 4.6), but the difference was not statistically significant (P = 0.29). The octogenarian patients presented a lower number of dual‐coil ICD leads (2.1% vs.4.8%, p = .019) and fewer coronary sinus leads (2.4% vs.4.9%, p = .028).

The most frequent indication of TLE for both groups was device‐related infection (Table 3). Non‐infective indication was less frequent in octogenarians than in other adult patients (6.5% vs. 12.2%, p = .027). The total number of leads extracted per procedure was similar (1.7 ± 0.8 vs. 1.8 ± 0.8, p = 0.12) and the dwell time of the oldest extracted lead was comparable (6.7 ± 5.8 vs. 7.0 ± 6.5, p = 0.56) between the two groups. The majority of the patients in both groups underwent transvenous lead extraction through the femoral approach. We did not observe any differences between the groups in the use of extraction tools. Complete procedure success rates were high and similar in octogenarians and younger patients (96.7% vs.95.3%, p = 0.39). No differences in the rates of major and minor complications were found between octogenarians and younger patients. All complications and deaths were grouped together for each population to create a composite of adverse events, and this was further analyzed. There was no statistically significant difference with respect to composite adverse events between groups (p = 0.74).

**TABLE 3:** Procedure data and outcomes

	Age < 80 (*n* = 922)	Age > 80 (*n* = 184)	p value
Indication			.027
Infectious	810 (87.8)	172 (93.5)	
Noninfectious	112 (12.2)	12 (6.5)	
General anesthesia	92 (10.0)	14 (7.6)	0.47
Number of leads extracted per procedure	1.7 ± 0.8	1.8 ± 0.8	0.12
Dwell time of the oldest extracted lead, year	6.7 ± 5.8	7.0 ± 6.5	0.56
Techniques of extraction			
Manual traction	263 (28.5)	44 (23.9)	0.20
Mechanical sheaths	48 (5.2)	5 (2.7)	0.15
Laser sheaths	43 (4.7)	11 (5.9)	0.45
Femoral access	568 (61.6)	124 (67.5)	0.14
Radiological success	897 (97.3)	180 (97.8)	0.86
Procedure outcomes			
Complete procedural success	879 (95.3)	178 (96.7)	0.39
Clinical success	909 (98.6)	182 (98.9)	0.73
Failure	13 (1.4)	2 (1.1)	0.73
Complications			
Major (other than death)	10 (1.1)	2 (1.1)	0.68
Minor	18 (1.9)	3 (1.6)	0.99
Deaths			
In the intraoperative period	2 (0.2)	1 (0.5)	0.98
Postoperatively within 30 days	3 (0.3)	1 (0.5)	0.82
Composite adverse events	40 (4.3)	7 (3.8)	0.74
Duration of hospitalization, day	23 ± 16	32 ± 18	<.001

Major complications occurred in 10 non‐octogenarian patients (1.1%), with six cardiac tamponades treated with success with pericardiocentesis, one cardiac tamponade treated by a heart surgeon, one pulmonary embolism treated by surgical thrombectomy, one tricuspid valve laceration treated by surgical repair and one paradoxical embolism treated by thrombolytic therapy. Of the major complications mentioned above, one tricuspid valve laceration and one cardiac tamponade occurred in patients treated by laser sheath. The remaining eight major complications occurred in procedures through femoral approach with Needle's Eye Snare. Major complications occurred in two octogenarian patients (1.1%) with two cardiac tamponades treated with pericardiocentesis. These two cardiac tamponades were related to extraction of ventricular leads by Needle's Eye Snare. All of the patients with major complications, both in non‐octogenarian and octogenarian group, recovered without sequelae. (Table 3).

Among the non‐octogenarian patients, there were two intra‐procedural deaths. Both cases developed lacerations of the right atrium and died despite urgent surgical repair. In the octogenarian group, one intra‐procedural death occurred as a result of the right ventricle laceration. All the culprit leads, both in non‐octogenarian and octogenarian group, were extracted by Needle's Eye Snare. Three patients in the non‐octogenarian group died of the following causes within 30 days after TLE: one of multiple organ dysfunction syndrome, one of severe heart failure and one of septic shock. There was only one postoperative death in the octogenarian group, which was due to septic shock. There was no statistically significant difference in the proportion of intra‐procedural (p = .74) and post‐procedural (p = 0.12) deaths between groups.

The duration of hospitalization(days) in the elderly patients was longer than that in the non‐elderly patients (23 ± 16 vs. 32 ± 18 days, p < .001).

**FIGURE 1:**
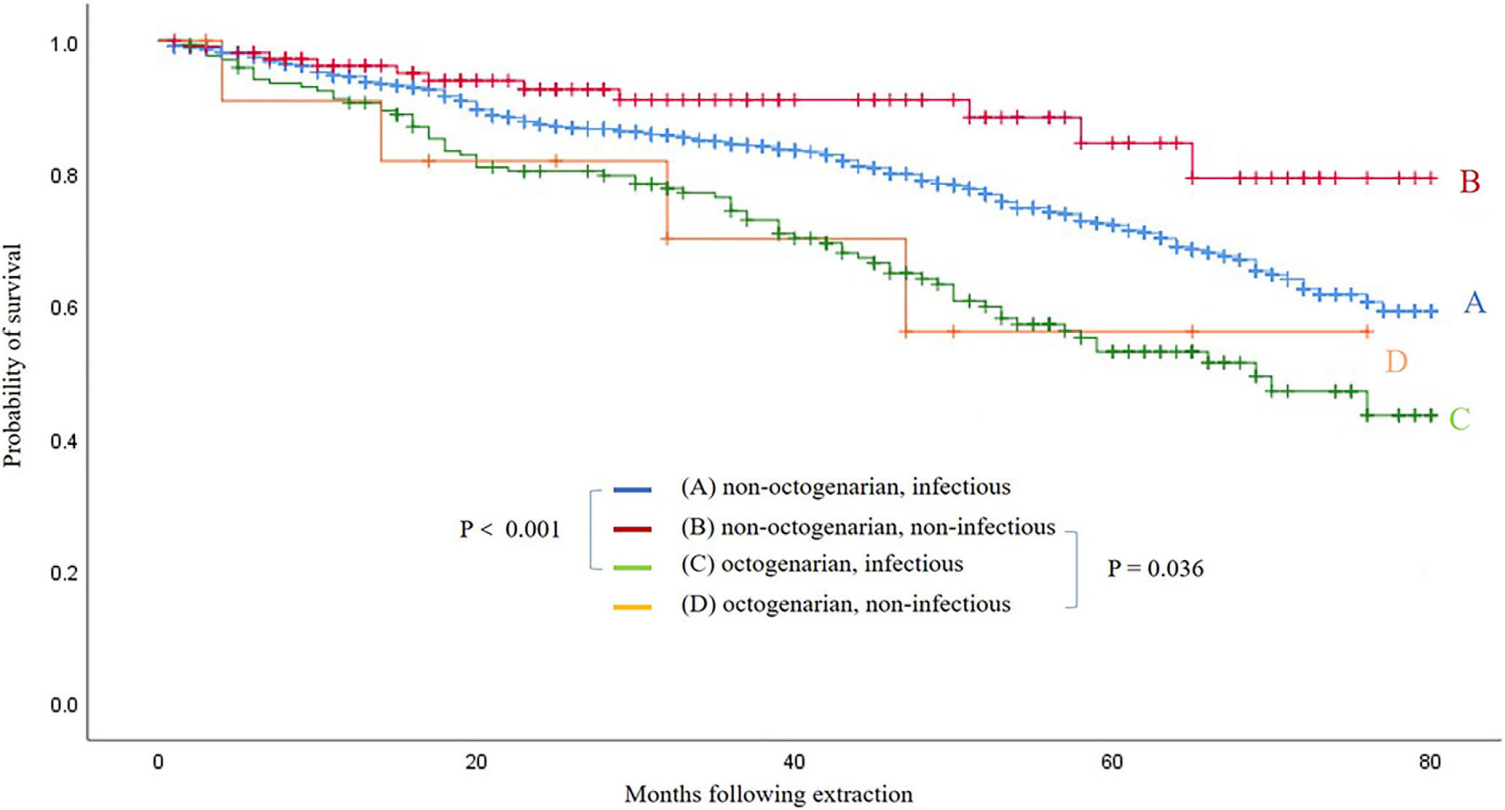
Overall Kaplan–Meier survival curves according to age and transvenous lead extraction indication

Patients were followed‐up to 7 years. The median follow‐up time was 3.3 years (IQR 1.5–6.0). Kaplan–Meier survival analysis of the octogenarian and non‐octogenarian group demonstrated that long‐term mortality was higher in octogenarian group and the difference was significant (p < .001). Survival data were further categorized according to lead extraction indication and Kaplan–Meier survival curves according to age and lead extraction indication were shown in Figure 1. Long‐term survival rate of octogenarians was poorer than that of non‐octogenarians in both the infectious and non‐infectious indication group (p < .001 and p = .036, respectively).(Figure 1).

## 5 DISCUSSION

This is a study describing the outcomes of TLE in a large cohort of patients from a single tertiary referral center in China. The major findings of this study are that advanced age and presence of comorbidities do not affect the safety and efficacy of transvenous lead extraction procedures in the Chinese population.

The clinical implications of our findings are potentially relevant. The elderly population is steadily increasing in China. Longer life expectancy prompts an increase of the number of CIED recipients, and consequently multiplies further reoperations. The rate of elderly patients presenting with device‐related infection or malfunction eligible for transvenous lead extraction is also expected to increase. Although TLE has been proven to be an effective and safe procedure, elderly patients with worse general population and more comorbidities are always under referral to this invasive procedure.^4^ In the single‐center analysis performed by Kennergren et al, an advanced age was one of the main reasons to discourage a TLE.^13^ In a survey of European electrophysiology centers, advanced age was independently associated with operator reluctance to perform transvenous lead extraction.^14^ In our analysis of a large cohort, the rate of clinical success was high, without significant differences between octogenarian and younger patients (98.9% vs 98.6%). This is in agreement with the literature, which demonstrated the safety and effectiveness of laser‐assisted lead extraction in the elderly population.^6^, ^15^, ^16^However, those studies enrolled only Caucasians, with BMI generally higher than that of the Asia population.^9^ And, average to small BMI has been proven to be an indicator of decreased clinical and procedural success.^17^ Notably, femoral approach with snares was the first‐line choice in our center, both for octogenarian and younger group (61.6% vs 67.5%), which was different from previous studies.

The octogenarians in our study had more comorbidities and higher CCI, whereas the rate of.

major complications was low and comparable with the younger patients. This could be influenced by the use of Needle's Eye Snare as a primary tool. Over 90% of the extracted leads were pacing leads. Bracke et al. reported that major complication rate was only 0.7% when extracting pacing leads by Needle's Eye Snare, which was comparable with the current study.^18^ In a study of 101 patients randomly assigned to extractions with a laser sheath (*n* = 50) versus a snare via femoral approach (*n* = 51), there were similar success and complication rates between the laser and femoral approach group.^19^

The most frequent major complication in both groups was cardiac tamponade due to lacerations of the right atrium or ventricle. Emergency pericardiocentesis can be effective in patients with cardiac tamponade; if unsuccessful, however, surgery must be rapidly performed. With the strategy of pericardiocentesis followed by a rescue surgical approach to treat cardiac tamponade, the ELECTRa study demonstrated a higher success rate of 93.8%.^20^ In the present study, most of the cardiac tamponades were relieved after pericardiocentesis (9/12,75%). However, death still occurred in three cardiac tamponades (3/12,25%) despite of urgent surgical repair.

Previous study showed no differences in long‐term survival between elderly and younger individuals.^6^ In their cohort, octogenarians actually had lower prevalence of coronary artery disease, diabetes, and higher ejection fraction compared to non‐octogenarians. This finding may be attributed by the fact that older patients with higher number of comorbidities may not receive CIED implantation in the first place. However, in this study we observed a significantly higher long‐term mortality rate among octogenarians than their counterparts, both for infectious and non‐infectious indication. High long‐term mortality rates are likely a reflection of the severity of the underlying disease process and associated comorbidities. It was consistent with the fact that octogenarians in our series had more comorbidities and higher CCI than younger patients and the differences were statistically significant.

## 6 LIMITATIONS

Our study is based on a retrospective analysis of data from consecutive patients referred to a tertiary center specialized in the CIED lead management and extraction. Because of referring doctors' discretion, some elderly patients with frailty or multiple comorbidities, were not referred to our center for the evaluation for lead extraction. As such, our data might be affected by referral bias.

Further, the retrospective design of our study might also act as a limitation. We could not be able to adjust the results by undetected or unretrievable confounders. Finally, all the procedures were performed by experienced operators, and the results may not be applicable to other lower procedural volume centers or less‐experienced operators.

## 7 CONCLUSIONS

Transvenous lead extraction through femoral approach in Chinese octogenarians has similar efficacy and safety compared to their younger counterparts. Despite the fact that octogenarians present more often with device‐related infection and have more comorbidities, the rates of complete procedural success and complications are comparable to younger patients. In this context, advanced age alone should not prevent candidacy for TLE.

## CONFLICT OF INTEREST

The authors declare no potential conflict of interest.

## Data Availability

The data that support the findings of this study are available from the corresponding author upon reasonable request.
